# Supplementary material for: A Systematic Review of Dynamic, Kinematic, and Muscle Activity during Gymnastic Still Rings Elements
Source: Sports (Basel). 2023 Feb 22;11(3):50. doi: 10.3390/sports11030050 (PMC10059656; doi:10.3390/sports11030050)
Supplement: Supplementary file 1 [file sports-11-00050-s001.zip › sports-2139499-supplementary.pdf]

Supplementary Material S1

Protocol of systematic review

1. Review title

A systematic review of dynamic, kinematic, and muscle activity during gymnastic still rings elements

2. Original language title.

A systematic review of dynamic, kinematic, and muscle activity during gymnastic still rings elements

3. Anticipated or actual start date.

01/12/2020

4. Anticipated completion date.

1/02/2023

5. Stage of review at time of this submission.

The review has started

6. Review stage Started Completed.

Preliminary searches: Yes

Piloting of the study selection process: Yes
Formal screening of search results against eligibility criteria: No
Data extraction: No
Risk of bias (quality) assessment: No
Data analysis: No

7. Named contact.

Roman Malíř
Mr Malir
malir@ftvs.cuni.cz

8. Named contact address.

Jose Martího 269/31, Prague 6 Veleslavín

9. Named contact phone number.

776335537

10. Organisational affiliation of the review.

Faculty of Physical education and Sport, Charles University
Organisation web address:
https://ftvs.cuni.cz/FTVSEN-1.html

11. Review team members and their organisational affiliations.

Roman Malir. Faculty of Physical education and Sport, Charles University
Jan Chrudimsky. Faculty of Physical education and Sport, Charles University
Michal Steffl. Faculty of Physical education and Sport, Charles University
Petr Stastny. Faculty of Physical education and Sport, Charles University

12. Funding sources/sponsors.

This review will be supported by the research grants of Charles University, Czech Republic [UNCE/HUM/032 and SVV 260599]

13. Conflicts of interest.

The authors declare no conflict of interest

14. Review question.

How can we evaluate and what are the appropriate evaluation methods and tools for assessing the performance of exercises on still rings within the framework of kinematics dynamics, and muscle work of swings; dismounts; handstands; strengths, and hold elements?

15. Searches.

The literature search will be implemented in four scientific databases: PubMed, EBSCO, Scopus, and Web of Science

16. URL to search strategy.

https://www.webofscience.com/wos/woscc/summary/1344d89c-6df3-4017-bed9-8b0601912fc0-6f4d851f/rele
vance/1 https://www.Scopus.com/results/results.uri?sort=plf-f&src=s&sid=e8f70683b52a8b9667556b1005f51a5b&sot
=a&sdt=a&sl=48&s=%28+TITLE-ABS-KEY+%28+gymnast*+%29+AND+ALL+%28+ring*+%29+%29&origin=

searchadvanced&editSaveSearch=&txGid=ed8de99ab28b6b236c2f525cac1ee443
https://PubMed.ncbi.nlm.nih.gov/?term=%28gymnast*%29+AND+%28ring*%29&sort=
https://web.p.EBSCOhost.com/ehost/resultsadvanced?vid=26&sid=4f911d7a-9ec0-4170-a78a-c878a095e26
7%40redis&bquery=TI+gymnast*+AND+ring*&bdata=JmRiPWFzbiZkYj1wZGgmZGI9cHN5aCZkYj1yZmgmZ
GI9bmxlYmsmZGI9bGxzJmRiPWx4aCZkYj1tc24mZGI9bWRjJmRiPXNpaCZkYj1zM2gmdHlwZT0xJnNlYXJj
aE1vZGU9U3RhbmRhcmQmc2l0ZT1laG9zdC1saXZl

17. Condition or domain being studied.

The domain of this systematic review will be the kinematic, dynamic, and muscle activity of specific elements on still rings.

18. Participants/population.

Specify the participants or populations being studied in the review. The preferred format includes details of both inclusion and exclusion criteria. Participants in this systematic review will be elite and sub-elite male gymnasts.

19. Intervention(s), exposure(s).

We will review interventions and cross-sectional measuring only in healthy male gymnasts. The inclusion criteria will be appropriate and valid procedures for data evaluation used in studies. Exclusion criteria will be any musculoskeletal or acute disease of participants included in the study or inappropriate procedure for data collection.

20. Comparator(s)/control.

Due to the heterogeneity of individual studies, we do not expect the possibility of comparing studies.

21. Types of study to be included.

We expect, that studies in this systematic review will include RCT, case studies, and cross-sectional studies without randomization.

22. Main outcome(s).

The aim of this review is to compile the dynamic, kinematic, and EMG characteristics of swing, dismount, handstand, strength, and hold elements on steady rings in any cross-sectional or intervention studies comparing any biomechanical measures on male gymnasts.. This review should be able to distinguish an appropriate training method, performance evaluation, and correct technique evaluation based on MAG CoP.

23. Additional outcome(s).

We assume that EMG will be a suitable tool for evaluating muscle work and muscle coordination at the strength and hold elements on still rings.

24. Data extraction (selection and coding).

We will use specific keywords within searching formula that will be used to find studies across databases. The Zotero personal research assistant will be used to arrange studies.

25. Risk of bias (quality) assessment.

We will assess these characteristics of each study: 1. Were the criteria for the inclusion of gymnasts clearly defined?
2. Were the gymnasts and the setting described in detail?
3. Was the exposure in valid and reliable way?
4. Were objective, standard criteria used for the measurement of conditions within the study?
5. Were confounding factors identified?
6. Were strategies to deal with confounding factors stated?
7. Were the outcomes of kinematic, dynamic, and EMG characteristics measured in a valid and reliable way?
8. Was appropriate statistical analysis used?

26. Strategy for data synthesis.

We will synthesize effect sizes, correlation coefficients, and other relevant results that will be substantiated within the collected studies.

27. Analysis of subgroups or subsets.

We expect that all participants will in this systematic review be only male gymnasts included. The potential rationale is for the investigation of subgroups within the age (young/adult) and level (elite/sub-elite).

28. Type and method of review.

Systematic review: Educational, Musculoskeletal problematic

29. Language.

English

30. Country.

Czech Republic

31. Current review status.

Data collection in process.

Supplementary Material S2

The JBI Checklist for Analytical Cross Sectional Studies

Study	Item 1	Item 2	Item 3	Item 4	Item 5	Item 6	Item 7	Item 8
Bango et. al. 2017, Spain, Observational study [8]	Y	Y	Y	NA	U	U	Y	Y
El-Awady, (2018), Egypt, Experimental study [31]	Y	Y	Y	NA	Y	Y	Y	Y
Sands, (2006), US, Observational study [13]	Y	Y	Y	NA	U	U	Y	Y
Bernasconi, (2004), France, Observational study [18]	Y	Y	Y	NA	Y	Y	Y	Y
Carrara, (2016), Brazil, Case study [43]	NA	Y	Y	NA	U	U	Y	Y
Dunlavy, (2007), US, Observational study [14]	Y	Y	Y	NA	U	U	Y	Y
Sands, (2006), US, Case study [17]	NA	N	Y	NA	N	N	Y	Y
Schärer, (2016), Switzerland, Observational study [32]	Y	Y	Y	NA	Y	Y	Y	Y
Hübner, (2015), Switzerland, Observational study [9]	Y	Y	Y	NA	Y	Y	Y	Y
Schärer, (2019), Switzerland, Quasi-experimental study [10]	Y	Y	Y	NA	Y	Y	Y	Y
Schärer, (2021), Switzerland, Quasi-experimental study [33]	Y	Y	Y	NA	Y	Y	Y	Y
Bango, (2013), Spain, Observational study [15]	Y	Y	Y	NA	U	U	Y	Y
Bernasconi, (2009), France, Observational study [1]	Y	Y	Y	NA	U	U	Y	Y
Gorosito, (2013), Argentina, Observational study [11]	Y	Y	Y	NA	Y	Y	Y	Y
Bernasconi, (2006), France, Observational study [16]	Y	Y	Y	NA	U	U	Y	Y
Khargan, (2020), Iraq, Experimental study [28]	Y	Y	Y	NA	U	U	Y	N
Yeadon, (2011), UK, Case study [29]	NA	Y	Y	NA	NA	NA	Y	Y
Irwin, (2002), UK, Observa-tional study [34]	Y	Y	Y	NA	U	U	U	U
Niu, (2000), China, Observational study [21]	Y	Y	Y	NA	U	U	Y	Y
Sprigings, (1997), Canada, Case study [23]	NA	Y	Y	NA	NA	NA	Y	NA
Sprigings, (2000), Canada, Case study [44]	NA	N	Y	NA	NA	NA	Y	NA
Yeadon, (2003), UK, Case study [5]	NA	Y	Y	NA	NA	NA	Y	NA
Brewin, (2000), UK, Case study [22]	NA	Y	Y	NA	NA	NA	Y	NA
Mills, (1998), UK, Case study [20]	NA	Y	Y	NA	NA	NA	Y	NA
Serafin, (2008), Poland, Case study [19]	NA	Y	Y	NA	NA	NA	Y	NA
Čuk, (2010), Slovenia, Case study [25]	NA	Y	Y	NA	NA	NA	Y	NA
Yeadon, (1994), UK, Observational study [24]	Y	N	N	NA	NA	NA	Y	Y
Ningxiang, (2012), China, Case study [26]	NA	Y	N	NA	NA	NA	Y	NA
Brewin, (2003), UK, Case study [7]	NA	Y	N	NA	NA	NA	Y	Y
Kochanowicz, (2019), Poland, Observational study [27]	Y	Y	Y	NA	Y	Y	Y	Y
Schärer, (2022), Switzerland, Quasi-experimental study [45]	Y	Y	Y	NA	NA	NA	Y	Y
Göpfer, (2022), Switzerland, Quasi-experimental study [49]	Y	Y	Y	NA	Y	Y	Y	Y
Lehmann, (2021), Germany, Observational study [50]	Y	Y	Y	NA	NA	NA	Y	Y
Fujihara, (2023), Japan, Observational study [54]	Y	U	Y	NA	NA	NA	Y	Y
Kolimechkov, (2021), UK, Bulgaria, Case study [46]	NA	Y	Y	NA	NA	NA	Y	Y
Goto, (2022), Brazil, Case study [47]	NA	U	Y	NA	NA	NA	NA	Y
Yanev, (2021), Bulgaria, Observational study [53]	Y	U	Y	NA	NA	NA	NA	Y

Y = yes scored as 1, N = no scored as 0, U = unclear, NA = not applicable.
1. Were the criteria for the inclusion of gymnasts clearly defined?
2. Were the gymnasts and the setting described in detail?
3. Was the exposure in valid and reliable way?
4. Were objective, standard criteria used for the measurement of conditions within the study?
5. Were confounding factors identified?
6. Were strategies to deal with confounding factors stated?
7. Were the outcomes of kinematic, dynamic, and EMG characteristics measured in a valid and reliable way?
8. Was appropriate statistical analysis used?

1. Bango, B.; Navandar, A.; Grande Rodríguez, I.; Quintana, M. Evaluation of Isometric Force Production in L-Sit Cross in Still Rings among Elite Male Artistic Gymnasts. *Journal of Human Sport and Exercise* **2017**, *12*. https://doi.org/10.14198/jhse.2017.122.02.
2. El-Awady, O. E.-E. Effects of Functional Suspension Training On L-Sit Cross in Still Rings Fro Egyptian Elite Male Artistic Gymnasts. *Ovidius University Annals, Series Physical Education and Sport/Science, Movement and Health* **2018**, *18* (1), 52–60.
3. Sands, W.; Stone, M.; Mcneal, J.; Smith, S. L.; Jemni, M.; Hewit, J.; Mizushima, K.; Haff, G. A Pilot Study to Measure Force Development during a Simulated Maltese Cross for Gymnastics Still Rings. In *XXIV International Symposium on Biomechanics in Sports*, Salzburg, Austria, 16th of July **2006**; p 407.
4. Bernasconi, S.; Tordi, N.; Parratte, B.; Rouillon, J. D. D.; Monnier, G. Surface Electromyography of Nine Shoulder Muscles in Two Iron Cross Conditions in Gymnastics. *J Sports Med Phys Fitness* **2004**, *44* (3), 240–245.
5. Carrara, P.; Amadio, A. C.; Serrão, J. C.; Irwin, G.; Mochizuki, L. The Cross on Rings Performed by an Olympic Champion. *Rev. bras. educ. fís. esporte* **2016**, *30*, 71–77. https://doi.org/10.1590/1807-55092016000100071.
6. Dunlavy, J. K.; Sands, W. A.; McNeal, J. R.; Stone, M. H.; Smith, S. L.; Jemni, M.; Haff, G. G. Strength Performance Assessment in a Simulated Men's Gymnastics Still Rings Cross. *J Sports Sci Med* **2007**, *6* (1), 93–97.
7. Sands, W.; Mcneal, J. The Inverted Cross: A Case Study with Training Implications. *Technique* **2006**, *26*, 22–23.
8. Schärer, C.; Hübner, K. Prediciton of Maximum Resistance Accuracy at Five and Seven Seconds Holding Times from a Three Seconds Static Maximum Strength Test of the Elements Iron Cross, Support Scale and Swallow on Rings Using the Devices Counterweight or Additional Weight. *Science of Gymnastics Journal* **2016**, *8*, 125–134.
9. Hübner, K.; Schärer, C. Relationship between Swallow, Support Scale and Iron Cross on Rings and Their Specific Preconditioning Strengthening Exercises. **2015**, *7*, 59–68.
10. Schärer, C.; Tacchelli, L.; Göpfert, B.; Gross, M.; Lüthy, F.; Taube, W.; Hübner, K. Specific Eccentric–Isokinetic Cluster Training Improves Static Strength Elements on Rings for Elite Gymnasts. *Int J Environ Res Public Health* **2019**, *16* (22), 4571. https://doi.org/10.3390/ijerph16224571.

11. Schärer, C.; Huber, S.; Bucher, P.; Capelli, C.; Hübner, K. Maximum Strength Benchmarks for Difficult Static Elements on Rings in Male Elite Gymnastics. *Sports (Basel)* **2021**, *9* (6), 78. https://doi.org/10.3390/sports9060078.
12. Bango, B.; Quintana, M.; Grande Rodríguez, I. New Tool to Assess the Force Production in the Swallow. *Science of Gymnastics Journal* **2013**, *5*, 47–58.
13. Bernasconi, S. M.; Tordi, N. R.; Parratte, B. M.; Rouillon, J.-D. R. Can Shoulder Muscle Coordination during the Support Scale at Ring Height Be Replicated during Training Exercises in Gymnastics? *J Strength Cond Res* **2009**, *23* (8), 2381–2388. https://doi.org/10.1519/JSC.0b013e3181bac69f.
14. Gorosito, M. A. Relative Strength Requirement for Swallow Element Proper Execution: A Predictive Test. *Science of Gymnastics Journal* **2013**, *5*, 59–67.
15. Bernasconi, S. M.; Tordi, N. R.; Parratte, B. M.; Rouillon, J.-D. R.; Monnier, G. G. Effects of Two Devices on the Surface Electromyography Responses of Eleven Shoulder Muscles during Azarian in Gymnastics. *J Strength Cond Res* **2006**, *20* (1), 53–57. https://doi.org/10.1519/R-16174.1.
16. Khargan, A. A.; Muhammad1, S. Y.; Alwan1, H. A. S. The Effect of Special Exercises by Using Training Methods to Assist in the Development of Some Motor Abilities and Handstand Skill of Still Rings for Young People. *Medico Legal Update* **2020**, *20* (1), 1211–1216. https://doi.org/10.37506/mlu.v20i1.539.
17. Yeadon, M. R.; Rosamond, E. L.; Hiley, M. J. The Biomechanical Design of a Gymnastics Training Aid for a Handstand on the Rings. *Proceedings of the Institution of Mechanical Engineers, Part P: Journal of Sports Engineering and Technology* **2011**, *226* (1), 24–31. https://doi.org/10.1177/1754337111424682.
18. Irwin, G.; Glazier, P.; Tong, R.; Radway, J. Changes in Muscle Activation Characteristics during Gymnastics Rings Routines. *Journal of Sports Sciences* **2002**, *20*, 10–11.
19. Niu, J.; Lu, X.; Xu, G.; Liang, B. Study on Gymnastics Ring Movements Using Force Measuring System. *ISBS - Conference Proceedings Archive.* Hong Kong, China. 25th – 30th June **2000**.
20. Sprigings, E. J.; Lanovaz, J. L.; Glen Watson, L.; Russell, K. W. Removing Swing from a Handstand on Rings Using a Properly Timed Backward Giant Circle: A Simulation Solution. *Journal of Biomechanics* **1997**, *31* (1), 27–35. https://doi.org/10.1016/S0021-9290(97)00110-3.
21. Sprigings, E.; Lanovaz, J.; Russell, K. The Role of Shoulder and Hip Torques Generated during a Backward Giant Swing on Rings. *Journal of Applied Biomechanics* **2000**, *16*, 289–300. https://doi.org/10.1123/jab.16.3.289.
22. Yeadon, M. R.; Brewin, M. A. Optimised Performance of the Backward Longswing on Rings. *Journal of Biomechanics* **2003**, *36* (4), 545–552. https://doi.org/10.1016/S0021-9290(02)00423-2.
23. Brewin, M. A.; Yeadon, M. R.; Kerwin, D. G. Minimising Peak Forces at the Shoulders during Backward Longswings on Rings. *Human Movement Science* **2000**, *19* (5), 717–736. https://doi.org/10.1016/S0167-9457(00)00033-6.
24. Mills, S. H. Indirect Measurement of Forces on the Gymnastics Rings. *ISBS - Conference Proceedings Archive* Konstanz, Germany. 21st -25th of July **1998**.
25. Serafin, R.; Golema, M.; Siemieński, A. Mechanical Loading of the Gymnast's Motor Systém During Swings on Rings. **2008**, 10.
26. Čuk, I.; Držaj, S.; Karacsony, I. Triple Salto Backward Tucked from Rings (a Case Study). / Trojni Skrčeni Salto Nazaj S Krogov (Študij Primera). *Kinesiologia Slovenica* **2010**, *16* (3), 23–32.
27. Yeadon, M. Twisting Techniques Used in Dismounts from Rings. *Journal of applied biomechanics* **1994**, *10*. https://doi.org/10.1123/jab.10.2.178.

28. Ningxiang, Z.; Mingxin, G.; Lejun, W.; Jiangbo, W. A Kinematic Analysis of Yan Mingyong's Landing from Rings after the Backward Giant Swing Tuck of 2-Circle Back Flip and a 360° Turn. *2012 International Conference on Systems and Informatics (ICSAI2012), Systems and Informatics (ICSAI), 2012 International Conference on* **2012**, 1050–1053. https://doi.org/10.1109/ICSAI.2012.6223213.
29. Brewin, M. A.; Kerwin, D. G. Indirect Estimation of Cable Tension during Gymnastic Movements on Rings. *Sports Eng* **2003**, *6* (3), 177–185. https://doi.org/10.1007/BF02859894.
30. Kochanowicz, A.; Niespodziński, B.; Mieszkowski, J.; Marina, M.; Kochanowicz, K.; Zasada, M. Changes in the Muscle Activity of Gymnasts During a Handstand on Various Apparatus. *Journal of Strength and Conditioning Research* **2019**, *33* (6), 1609–1618. https://doi.org/10.1519/JSC.0000000000002124.
31. Schärer, C.; Bucher, P.; Lüthy, F.; Hübner, K. Combined Eccentric-Isokinetic and Isoinertial Training Leads to Large Ring-Specific Strength Gains in Elite Gymnasts. *Sports (Basel)* **2022**, *10* (4), 49. https://doi.org/10.3390/sports10040049.
32. Kolimechkov, S.; Yanev, I.; Kiuchukov, I.; Petrov, L. Kinematic Analysis of Double Back Straight Somersault and Double Back Straight Somersault with Full Twist on Rings. *Sci. Gymnast. J.* **2021**, *13* (2), 191–202. https://doi.org/10.52165/sgj.13.2.191-202.
33. Goto, M.; Carrara, P.; Lopes, H.; Nunomura, M. Towards the Olympic Gold: The Training Plan on the Gymnastics Rings. *Sci. Gymnast. J.* **2022**, *14* (2), 145–158. https://doi.org/10.52165/sgj.14.2.145-157.
34. Göpfert, B.; Schärer, C.; Tacchelli, L.; Gross, M.; Lüthy, F.; Hübner, K. Frequency Shifts in Muscle Activation during Static Strength Elements on the Rings before and after an Eccentric Training Intervention in Male Gymnasts. *J Funct Morphol Kinesiol* **2022**, *7* (1), 28. https://doi.org/10.3390/jfmk7010028.
35. Lehmann, T.; Winter, A.; Seemann-Sinn, A.; Naundorf, F. Use of Objective Methods to Determine the Holding Time of Hold Elements on Still Rings. *Sci. Gymnast. J.* **2021**, *13* (2), 181–189. https://doi.org/10.52165/sgj.13.2.181-189.
36. Yanev, I. Trends of Exercises Content on the Rings Final from the 1st Junior World Artistic Gymnastics Championships. *Trakia Journal of Sciences* **2021**, *19*, 847–852. https://doi.org/10.15547/tjs.2021.s.01.133.
37. Fujihara, T. Real-Time Video and Force Analysis Feedback System for Learning Strength Skills on Rings in Men's Artistic Gymnastics. *Sports Biomech* **2023**, *22* (2), 186–194. https://doi.org/10.1080/14763141.2021.2024873.

Supplementary Material S3

The PRISMA checklist

Section and Topic	Item #	Checklist item	Location where item is reported
**TITLE**			
Title	1	Identify the report as a systematic review.	Page 1, Lines1-3
**ABSTRACT**			
Abstract	2	See the PRISMA 2020 for Abstracts checklist.	Page 1, Lines 7-20
**INTRODUCTION**			
Rationale	3	Describe the rationale for the review in the context of existing knowledge.	Page 1-2, Lines 24-93
Objectives	4	Provide an explicit statement of the objective(s) or question(s) the review addresses.	Page 2, Lines 87-93
**METHODS**			
Eligibility criteria	5	Specify the inclusion and exclusion criteria for the review and how studies were grouped for the syntheses.	Page 4, Lines 161-167
Information sources	6	Specify all databases, registers, websites, organisations, reference lists and other sources searched or consulted to identify studies. Specify the date when each source was last searched or consulted.	Page 3, Lines 122-123
Search strategy	7	Present the full search strategies for all databases, registers and websites, including any filters and limits used.	Page 4, Lines 180-182
Selection process	8	Specify the methods used to decide whether a study met the inclusion criteria of the review, including how many reviewers screened each record and each report retrieved, whether they worked independently, and if applicable, details of automation tools used in the process.	Page 4, Lines 161-167
Data collection process	9	Specify the methods used to collect data from reports, including how many reviewers collected data from each report, whether they worked independently, any processes for obtaining or confirming data from study investigators, and if applicable, details of automation tools used in the process.	Page 3, Lines 104-118
Data items	10a	List and define all outcomes for which data were sought. Specify whether all results that were compatible with each outcome domain in each study were sought (e.g. for all measures, time points, analyses), and if not, the methods used to decide which results to collect.	Not applied
	10b	List and define all other variables for which data were sought (e.g. participant and intervention characteristics, funding sources). Describe any assumptions made about any missing or unclear information.	Not applied
Study risk of bias assessment	11	Specify the methods used to assess risk of bias in the included studies, including details of the tool(s) used, how many reviewers assessed each study and whether they worked independently, and if applicable, details of automation tools used in the process.	Page 4, Lines 176-177
Effect measures	12	Specify for each outcome the effect measure(s) (e.g. risk ratio, mean difference) used in the synthesis or presentation of results.	Not applied
Synthesis methods	13a	Describe the processes used to decide which studies were eligible for each synthesis (e.g. tabulating the study intervention characteristics and comparing against the planned groups for each synthesis (item #5)).	Page 4, Lines 161-167
	13b	Describe any methods required to prepare the data for presentation or synthesis, such as handling of missing summary statistics, or data	Not applied

Section and Topic	Item #	Checklist item	Location where item is reported
		conversions.	
	13c	Describe any methods used to tabulate or visually display results of individual studies and syntheses.	Not applied
	13d	Describe any methods used to synthesize results and provide a rationale for the choice(s). If meta-analysis was performed, describe the model(s), method(s) to identify the presence and extent of statistical heterogeneity, and software package(s) used.	Page 4, Lines 169-177
	13e	Describe any methods used to explore possible causes of heterogeneity among study results (e.g. subgroup analysis, meta-regression).	Not applied
	13f	Describe any sensitivity analyses conducted to assess robustness of the synthesized results.	Not applied
Reporting bias assessment	14	Describe any methods used to assess risk of bias due to missing results in a synthesis (arising from reporting biases).	Page 4, Lines 176-177
Certainty assessment	15	Describe any methods used to assess certainty (or confidence) in the body of evidence for an outcome.	Not applied
**RESULTS**			
Study selection	16a	Describe the results of the search and selection process, from the number of records identified in the search to the number of studies included in the review, ideally using a flow diagram.	Page 4, Lines 183-201 (Figure 1)
	16b	Cite studies that might appear to meet the inclusion criteria, but which were excluded, and explain why they were excluded.	Not applied
Study characteristics	17	Cite each included study and present its characteristics.	Page 5-14, Tables 2-5
Risk of bias in studies	18	Present assessments of risk of bias for each included study.	Page 4, Lines 176-177
Results of individual studies	19	For all outcomes, present, for each study: (a) summary statistics for each group (where appropriate) and (b) an effect estimate and its precision (e.g. confidence/credible interval), ideally using structured tables or plots.	Page 5-14, Tables 2-5
Results of syntheses	20a	For each synthesis, briefly summarise the characteristics and risk of bias among contributing studies.	Page 4, Lines 176-177
	20b	Present results of all statistical syntheses conducted. If meta-analysis was done, present for each the summary estimate and its precision (e.g. confidence/credible interval) and measures of statistical heterogeneity. If comparing groups, describe the direction of the effect.	Not applied
	20c	Present results of all investigations of possible causes of heterogeneity among study results.	Page 4, Lines 190-201
	20d	Present results of all sensitivity analyses conducted to assess the robustness of the synthesized results.	Not applied
Reporting biases	21	Present assessments of risk of bias due to missing results (arising from reporting biases) for each synthesis assessed.	Page 4, Lines 176-177
Certainty of evidence	22	Present assessments of certainty (or confidence) in the body of evidence for each outcome assessed.	Not applied

Section and Topic	Item #	Checklist item	Location where item is reported
**DISCUSSION**			
Discussion	23a	Provide a general interpretation of the results in the context of other evidence.	Page 14-19, Lines 417-1032
	23b	Discuss any limitations of the evidence included in the review.	Not applied
	23c	Discuss any limitations of the review processes used.	Not applied
	23d	Discuss implications of the results for practice, policy, and future research.	Page 19, Lines 1014-1015
**OTHER INFORMATION**			
Registration and protocol	24a	Provide registration information for the review, including register name and registration number, or state that the review was not registered.	Page 2, Line 98
	24b	Indicate where the review protocol can be accessed, or state that a protocol was not prepared.	Page 4, Lines 180-182
	24c	Describe and explain any amendments to information provided at registration or in the protocol.	Not applied
Support	25	Describe sources of financial or non-financial support for the review, and the role of the funders or sponsors in the review.	Page 21, Lines 474-475
Competing interests	26	Declare any competing interests of review authors.	Page 19, Line 1045
Availability of data, code and other materials	27	Report which of the following are publicly available and where they can be found: template data collection forms; data extracted from included studies; data used for all analyses; analytic code; any other materials used in the review.	Page 4, Lines 180-182

Supplementary Material S4

Full seacrh strategy of all databases

Search PubMed:
https://pubmed.ncbi.nlm.nih.gov/?term=%28gymnast*%29+AND+%28ring*%29&sort
Key: "gymnast*"[All Fields] AND "ring*"[All Fields]
Methodology: all sources
Filter: No filter applicated
Seacrh period: 1961-2023
Date of search: 06/02/2023

Search EBSCOhost:
https://web.s.ebscohost.com/ehost/resultsadvanced?vid=3&sid=026cae85-b3ec-4287-8cf3-1641e24dcfef%40redis&bquery=TI+gymnast*+AND+ring*&bdata=JmRiPWFzbiZkYj1wZGgmZGI9cHN5aCZkYj1yZmgmZGI9bmxlYmsmZGI9bGxzJmRiPWx4aCZkYj1tc24mZGI9bWRjJmRiPXNpaCZkYj1zM2gmdHlwZT0xJnNlYXJjaE1vZGU9U3RhbmRhcmQmc2l0ZT1laG9zdC1saXZl
Key: (TI gymnast*) AND (ring*)
Methodology: all sources
Filter: No filter applicated
Seacrh period: 1940-2023
Date of search: 06/02/2023

Search Scopus:
https://www.scopus.com/results/results.uri?sort=plf-f&src=s&sid=6d097f0e877e78f00c3b7d1bcbb2dd7d&sot=a&sdt=a&sl=38&s=TITLE-ABS-KEY%28gymnast*%29+AND+ALL%28ring*%29&origin=searchadvanced&editSaveSearch=&txGid=09b7cbb4fd71fcd769a777606ab3c5fa
Key: (TITLE-ABS-KEY (gymnast*) AND ALL (ring*))
Methodology: all sources
Filter: No filter applicated
Seacrh period: 1961-2023
Date of search: 06/02/2023

Search Web of Science:
https://www.webofscience.com/wos/woscc/summary/11f519a0-98e9-4bd4-8596-a5f911b930c6-701bc572/relevance/1
Key: TOPIC: (gymnast*) AND TOPIC: (ring*)

Methodology: all sources
Filter: No filter applicated
Seacrh period: 1974-2022
Date of search: 06/02/2023
